# Comparative Analysis of Lower Limb Impairment Ratings in the AMA Guides Sixth Edition 2024 vs. 2008: Implications for Stakeholders

**DOI:** 10.3390/jcm14197033

**Published:** 2025-10-04

**Authors:** J. Mark Melhorn, Barry Gelinas, Douglas W. Martin, Kurt T. Hegmann, Matthew S. Thiese

**Affiliations:** 1 Department of Orthopaedics, University of Kansas School of Medicine Wichita, Wichita, KS 67214, USA; 2 International Academy of Independent Medical Evaluators, Vancouver, WA 98683, USA; barry@bgelinasmddc.com; 3 CNOS Occupational Medicine, Dakota Dunes, SD 57049, USA; douglaswaynemartin7@gmail.com; 4 Rocky Mountain Center for Occupational and Environmental Health, University of Utah, Weber State University, Salt Lake City, UT 84112, USA; kurt.hegmann@hsc.utah.edu (K.T.H.); matt.thiese@hsc.utah.edu (M.S.T.)

**Keywords:** AMA, impairment, efficiency, accuracy, validity reliability, lower limb

## Abstract

**Background/Objectives**: This study examines the effect of the 2024 update to the *AMA Guides to the Evaluation of Permanent Impairment*, Sixth Edition, on lower limb impairment determinations in comparison to the 2008 edition. It also explores the broader influence of these changes on regulatory, economic, and adjudicative considerations relevant to physician application and interpretation. **Methods:** Two experienced evaluators independently reviewed 23 standardized lower limb case scenarios, applying both the 2008 and 2024 methodologies. Each assessment was based solely on clinical history, physical examination findings, and diagnostic test results. Impairment values were then calculated and analyzed for consistency across editions. **Results:** The 2024 lower limb impairment framework produced outcomes that closely mirrored those of the 2008 edition, with intraclass correlation coefficients of 0.9962 for the lower limb and 0.9951 for whole-person impairment, underscoring the strong consistency between editions. **Conclusions:** The revised 2024 edition for lower limb assessment enhances procedural clarity and integrates improved diagnosis-based impairment tools without disrupting prior impairment values. These refinements are intended to improve utility for clinical and nonclinical stakeholders, ensuring reliable evaluations while minimizing systemic disruption.

## 1. Introduction

The *AMA Guides to the Evaluation of Permanent Impairment (AMA Guides)* serve as the global benchmark for consistent, evidence-based impairment assessment [1,2,3,4,5]. In the United States, workers’ compensation insurance—one of the primary systems that relies on impairment ratings—typically represents about 1% of payroll, equating to approximately $500 to $1200 per employee annually [6]. Nationally, this equates to an estimated $55 to $60 billion in annual insurance premiums [7]. When broader economic factors are considered, including medical costs, wage replacement, and lost productivity, the total cost of work-related injuries reached approximately $176.5 billion in 2023 [8]. Impairment ratings are critical for translating clinical observations into a quantifiable deviation, loss, or loss of use of any body structure or function in an individual with a health condition, disorder, or disease. The typical process begins by confirming a diagnosis once the individual has reached maximum medical improvement, followed by assigning a value that represents the degree of functional impairment. An accurate and equitable impairment determination is essential, as it provides the basis for those responsible for calculating disability-related benefits. Final compensation outcomes are usually established within judicial or administrative workers’ compensation systems and are influenced by statutory requirements, policy decisions, and broader societal considerations [1,2,3,4,5]. This article focuses exclusively on lower extremity impairments, highlighting the methodological refinement implemented in the 2024 edition of the *AMA Guides* compared to the 2008 version, and examining their impact on the impairment evaluation process. To address concerns raised about previous editions, such as limited testing for reliability, precision, and validity, which contributed to a challenging learning curve [9,10,11,12,13,14], the AMA established the Guides Editorial Panel in June 2019, which subsequently formed the musculoskeletal subcommittee in August 2022 to lead a thorough and coordinated revision process. Utilizing the RAND/UCLA modified Delphi Appropriateness Method and integrating organized input from the public, these efforts culminated in the 2024 edition of the AMA Guides. This updated version introduces a new standardized multi-step protocol for performing impairment evaluations and significantly enhances the diagnosis-based impairment (DBI) tables (Table 1). The revised DBI tables utilize specific individual elements obtained from clinical history, physical examination, and relevant clinical studies, each linked to defined criteria within the DBI tables.

The AMA Guides to the Evaluation of Permanent Impairment serve as the international standard for determining impairment ratings, with significant implications for clinical practice, workers’ compensation, disability adjudication, and insurance systems. Since the release of the Sixth Edition in 2008, questions have persisted regarding consistency, transparency, and the practical application of impairment ratings. In 2024, a major update to the Sixth Edition introduced methodological refinements aimed at enhancing clarity, usability, and alignment with current medical practice. Despite these improvements, little is known about how the 2024 revisions affect the determination of impairment values compared to the 2008 edition. Given the regulatory, economic, adjudicative, and patient-centered importance [15] of consistent ratings, it is essential to evaluate whether the updated framework alters impairment outcomes in a meaningful way.

The primary research question is: Does the AMA Guides Sixth Edition 2024 produce lower limb impairment values consistent with those of the 2008 edition, based on assessments by expert evaluators, and what are the implications of any differences for physicians, individuals in the context of patient-centered management, and other judicial, legislative, and economic stakeholders? If the impairment values remain stable across editions, the transition to the 2024 edition—with its added clarity, transparency, and functionality—may result in minimal disruption for evaluators and stakeholders; however, notable discrepancies could introduce challenges for physicians, as well as judicial and legislative entities tasked with implementing or interpreting impairment values.

## 2. Materials and Methods

This investigation analyzed impairment ratings through the use of standardized clinical vignettes. Because no human subjects were directly involved, the RedCap Institutional Review Board at the University of Kansas Medical Center designated the project as a quality improvement activity rather than human subjects research. We complied with the applicable Strengthening the Reporting of Observational Studies in Epidemiology Statement [16].

### 2.1. Vignettes and Diagnoses

This study utilized 19 lower extremity diagnosis-based impairment (DBI) vignettes sourced from the 2008 edition of the *AMA Guides*, Sixth Edition, supplemented by an additional example, “Amputation of Great Toe” (*AMA Guides*, 6th ed. 2008, p. 552), bringing the total to 23 vignettes. These 2008 vignettes were designed to enhance the consistency of impairment evaluations by providing step-by-step guidance for specific diagnoses. They were selected for the study because they represent quintessential examples of the 2008 impairment rating method.

Table 2 Vignette Number and Associated Diagnosis illustrates the range of diagnostic conditions of the lower limb commonly encountered in impairment evaluations, providing insight into the clinical diversity represented across vignettes.

### 2.2. Impairment Assessment

Impairment ratings were conducted by two senior physician members of the Guides Panel, whose combined expertise in musculoskeletal impairment assessment exceeds 50 years. They performed impairment assessments using both the 2008 and 2024 methodologies, applying each to the same set of 23 standardized vignettes. For every assessment, the evaluators were provided solely with the three core elements of the diagnostic process—clinical history, physical findings, and study results from the vignettes—without disclosure of predetermined impairment values.

Initially, the evaluators applied the 2008 methodology to each vignette, resulting in impairment values that matched those published in the *AMA Guides* Sixth Edition 2008. They then reassessed the same vignettes using the updated 2024 methodology, which incorporates revised stepwise assessment framework and enhanced DBI tables. A repeat assessment was conducted after an 8-week interval, during which both the 2008 and 2024 editions were reapplied, with vignettes redistributed in a newly randomized sequence for each evaluator. The interval was deliberately chosen to limit recall bias from the initial round.

### 2.3. Outcome Measure

The principal outcomes assessed were lower limb impairment and whole person impairment, expressed as percentages of functional loss according to the standardized scale of the AMA Guides [17]. The two evaluators calculated the lower limb impairment and whole person impairment for each of the 23 vignettes using both the 2008 and updated 2024 methodology. A comparative analysis was performed to assess the differences between the impairment values generated with the 2008 methodologies and those generated with the 2024 methodologies. The Wilcoxon signed-rank test, a nonparametric statistical test, was used to determine the significance of the differences in median impairment values, and *p* values for the differences were calculated. We also used the Exact test, a nonparametric test that is useful for very small samples when analyzing differences for lower limb impairment values. Interclass correlation coefficient was calculated for the reliability between the 2008 and 2024 methodologies for both the lower limb and whole person impairment values.

After an eight-week interval, the evaluators repeated the assessments, with the vignettes presented in a newly randomized sequence to test consistency. This design allowed for a systematic comparison of the 2024 AMA Guides methodology with the 2008 edition for both lower limb and whole person impairment values. Statistical methods were used to measure the extent and significance of any differences between the two rating systems, offering a comprehensive evaluation of the updates. All statistical analyses were conducted at a significance threshold of 0.05 using SAS software, version 9.4 (SAS Institute, Cary, NC, USA) [18].

## 3. Results

### 3.1. Impairment Rating

The final lower limb impairment values assigned to all 23 vignettes were consistent across both rounds for each evaluator when using the 2024 methodology. This resulted in a Cohen’s Kappa coefficient of 1.00, calculated with SAS, reflecting perfect inter-rater agreement while accounting for chance [19,20]. A value of 1.00 confirms that both evaluators independently arrived at identical impairment values for every vignette in both assessments, underscoring the reliability, consistency, and reproducibility of the updated 2024 approach when applied to a standardized clinical dataset. These findings offer compelling support for the methodological stability of the 2024 lower limb evaluation process, with minimal variability observed between experienced evaluators. The uniformity in results across repeated assessments reinforces confidence in the dependability of the impairment values derived using the revised guidelines.

### 3.2. Comparison of 2008 and 2024 Lower Limb Impairment Ratings

The impairment values determined by the evaluators using both the 2024 and 2008 methodologies are presented in Table 3.

For the lower limb impairment values across the 23 vignettes:

- 17 of 23 vignettes (74%) yielded identical impairment ratings across the two approaches.
- For the remaining six vignettes:
- Three vignettes demonstrated slightly elevated impairment values when assessed with the 2024 approach.
- Three vignettes demonstrated reduced impairment values when evaluated with the 2024 approach.

The aggregated mean lower limb impairment score for the 23 vignettes was identical—18.17—when calculated with either the 2024 or 2008 methodology, yielding a net difference of 0.00.

For the whole person impairment values across the 23 vignettes:

- 18 of 23 (78%) vignettes had the same whole person impairment values between the two methods.
- For the remaining five vignettes:
- Three vignettes had a slightly higher impairment value with the 2024 method;
- Two vignettes had a slightly lower impairment value for the 2024 method.

The combined average whole person impairment value across all 23 vignettes was 7.33 with the 2024 method and 7.29 with the 2008 method. This represents a 0.04 difference in the average impairment value between the 2008 and 2024 methodologies.

### 3.3. Impairment Averages Between Expert Evaluators

To provide a wider perspective on the impact of the revisions between the 2008 and 2024 *AMA Guides*, impairment values were combined across two categories: lower limb and whole person. This aggregation supports a more comprehensive understanding of the variations observed between the editions, as illustrated in Table 4.

When evaluating the impairments collectively, the average rating was 12.73% with the 2008 method and 12.75% with the 2024 method (*p* = 0.92) (Table 4). The interclass correlation coefficients for the lower limb and whole person were 0.9962 and 0.9951, respectively.

This comparison indicates that, while there are some variations in impairment values between the two *AMA Guides* editions, the changes are relatively minor. For the lower limb impairment values, 74% of the vignettes had the same ratings, with the remaining 26% showing only small differences of 1% to 5%. The average lower limb impairment value across all vignettes did not differ between the 2024 and 2008 methodologies.

For whole person impairment, 78% of vignettes produced identical ratings, while the remaining 22% differed only slightly (1–2%). The mean whole person value varied by just 0.04 between editions.

The present analysis provides useful understandings into the broader impact of the methodological updates introduced in the 2024 *AMA Guides* compared with the previous 2008 edition. The strong consistency observed in impairment values, with only minimal differences, indicates that the 2024 updates have generally preserved the continuity of the evaluation process while introducing refinements aimed at providing clarity and accuracy.

Overall, these data indicate that the transition from the 2008 *AMA Guides* to the 2024 edition is likely to have a modest impact on impairment values in clinical practice, based on the vignettes evaluated in this study.

## 4. Discussion

This study centers on selected lower limb conditions as listed in Table 2 Vignette Number and Associated Diagnosis and demonstrates that the results align with previous evaluations of upper limb and spine and pelvis impairments, showing consistency in impairment values between the 2008 and 2024 *AMA Guides* methodologies [21,22,23,24,25]. The 2024 edition preserves consistency in impairment values while enhancing clarity, usability, and transparency. These improvements are particularly valuable for physician evaluators, as the refined diagnostic processes and updated DBI tables reduce complexity, ease training demands, and support multiple diagnostic presentations—contributing to improved interrater and intrarater consistency. Collectively, these changes promote more reproducible evaluations and offer reassurance that the transition will streamline workflows without disrupting adjudication or compensation systems. These results further imply that adoption of the 2024 edition could alleviate some of the strain and discontent previously documented in workers’ compensation contexts [10,14,26,27,28,29].

The 2024 update to the AMA Guides, Sixth Edition, introduces a diagnostic row–based framework that is more intuitive and clinically relevant than the 2008 edition. In the 2008 model, evaluators often had to navigate a complex set of impairment grids and modifiers, which created opportunities for inconsistency and confusion. The 2024 approach streamlines this process by anchoring impairment determinations directly to a specific diagnostic row, allowing evaluators to begin with a clearly defined clinical diagnosis rather than reverse-engineering a category from abstract criteria.

This diagnostic row structure integrates three essential elements of medical evaluation: clinical history, physical examination, and relevant clinical studies. By linking impairment ratings directly to diagnoses that match the individual’s documented clinical presentation, the 2024 edition mirrors the way physicians already think and work in daily practice. For example, instead of broadly classifying “knee disorders,” the updated tables guide evaluators to identify the clinically relevant diagnosis, confirm it through physical findings, and corroborate it with imaging or other diagnostic studies where appropriate. This alignment reduces subjectivity, improves reproducibility, and ensures that the assigned impairment value reflects the real clinical condition.

As a result, the 2024 framework is easier to use, more transparent, and less prone to variability across evaluators. It allows physicians to apply the Guides in a way that parallels clinical reasoning, supporting both accuracy and efficiency. This shift enhances the consistency of impairment determinations, making the evaluation process more straightforward for physicians while also improving confidence among stakeholders—including patients, insurers, and adjudicators—that the ratings are evidence-based, clinically grounded, and reproducible.

A key finding of this study was the strong accuracy, consistency, reliability, and reproducibility demonstrated between the two physician evaluators for the 2024 process, evidenced by a Cohen’s Kappa of 1.00. The median lower limb impairment was identical across editions, and the median whole-person impairment showed only minimal differences, with most vignettes yielding the same impairment values in both 2008 and 2024. Furthermore, the 2024 lower limb impairment framework produced outcomes that closely mirrored those of the 2008 edition, with intraclass correlation coefficients of 0.9962 for the lower limb and 0.9951 for whole-person impairment. This high level of consistency confirms the equivalence of impairment determinations between the two versions. However, there were minor degrees of variability around the mean values, reflected in the slight differences in *p* values and interclass correlation coefficients. This high degree of concordance is likely to reduce stakeholder concerns regarding possible negative effects on impaired individuals and related economic outcomes.

To illustrate the practical benefits of transitioning from the 2008 to the 2024 AMA Guides Sixth Edition, Table 5 Comparison of the 2008 and 2024 AMA Guides Sixth Edition for Lower Limb Impairment summarizes the major differences in framework, clinical integration, usability, consistency, and stakeholder impact, with particular emphasis on the enhanced diagnostic row approach and its alignment with patient-centered management.

The AMA Guides are widely regarded as the international standard for objective, evidence-based impairment evaluation, integrating contemporary clinical and scientific knowledge [4,23,30]. The 2024 revisions were developed in response to stakeholder input, with particular emphasis on the musculoskeletal chapters addressing the upper limb, lower limb, and spine and pelvis. The enhanced DBI tables are designed to provide fair and equitable impairment values using the latest scientific advancements, building on prior methodologies. The revisions are intended to enhance usability, consistency, reliability, and reproducibility, while maintaining stability in impairment values. Because impairment evaluation is a multi-step process, maintaining transparency and reproducibility is essential to support accuracy and uphold confidence among workers’ compensation systems and legal stakeholders. Our comparative analysis suggests these goals have been achieved, with only minor implications for legal systems but significant benefits for evaluators in terms of improved clarity and ease of use. We acknowledge that while the *AMA Guides* provide an impairment value, the final determination of compensation or disability lies within the adjudicator’s jurisdiction.

This study has several limitations. Chief among them is the reliance on observational data derived from standardized case examples rather than real-life, face-to-face clinical encounters. Although hypothetical, the use of standardized cases from the 2008 edition ensured a consistent clinical dataset across evaluations, thereby enhancing comparability between methods and minimizing the risk of incomplete assessments resulting from variable patient histories. However, the relatively limited range of diagnoses and impairments—while representative of common clinical scenarios—may not fully capture the diversity of real-world presentations.

Future research should include broader investigations encompassing heterogeneous and more complex patient populations to provide deeper insight into real-world applicability, though such studies present substantial practical challenges. It should also be recognized that the impairment ratings in this analysis were derived from consensus and expert opinion. In clinical practice, individuals often exhibit diverse conditions, variable impairment severity for similar diagnoses, and intricate medical histories influenced by numerous contextual factors [31,32,33]. These complexities, coupled with the inherent variability in individual responses, underscore the difficulty of achieving fully standardized assessments in real-world settings.

While the use of only two expert evaluators has been suggested as a limitation, we respectfully note that in the context of this study, it is more accurately viewed as a strength. The primary objective was to compare impairment values between the 2008 and 2024 AMA Guides, and this comparison requires ratings that are accurate, consistent, and clinically sound. Experienced evaluators are best positioned to apply both editions correctly, minimizing variability and ensuring that any observed differences truly reflect changes in the Guides rather than differences in evaluator expertise.

To further reduce potential bias, the evaluators strictly adhered to the clinical data provided in the vignettes, which standardized the assessment process. While novice evaluators may produce different results, introducing that variability was not the intent of this study. Instead, our design ensured that differences in outcomes could be attributed to the editions themselves, thereby strengthening the validity of the findings.

Although the 2024 AMA Guides offer more detailed direction, atypical or complex clinical presentations may still require evaluator judgment to appropriately apply the rating framework. Such scenarios often involve a level of subjective interpretation, despite the structured nature of the updated criteria. These considerations reflect the ongoing challenges in impairment assessment and emphasize the importance of continued research to support the effective use of revised methodologies across a wide range of clinical situations.

It is essential to emphasize that the AMA Guides are designed to quantify impairment based on objective clinical findings, not to determine disability or compensation outcomes, as those decisions are reserved for the appropriate adjudicating authority [34,35,36,37,38,39]. Although the Guides focus exclusively on the objective evaluation of impairment, disability determinations consider a broader spectrum of factors—including age, functional capacity in daily activities, educational attainment, occupational demands, regional context, workplace accommodations, social support systems, and the overall impact on community participation—all of which extend beyond the scope of impairment assessment. Consequently, impairment ratings serve to inform, but do not solely determine, disability outcomes.

## 5. Conclusions

This comparative analysis confirms that the 2024 Sixth Edition of the AMA Guides preserves the foundational principles of impairment assessment while introducing refinements that enhance clinical applicability and administrative utility. Across 23 standardized lower extremity case scenarios, impairment values were nearly identical between the 2008 and 2024 methodologies, supporting both statistical and clinical equivalence. These results provide assurance to physicians, adjudicators, insurers, and policymakers that the revised approach maintains the validity and fairness of prior assessments.

The 2024 edition introduces a more structured and transparent methodology that improves diagnostic clarity, strengthens documentation, and supports reproducible outcomes. Key changes include a systematic framework for confirming medically appropriate diagnoses through integration of clinical history, physical examination, and relevant studies into the updated diagnosis-based impairment (DBI) tables. This process enables evaluators to consistently identify the correct diagnostic row, class, grade, and impairment value with greater precision.

Despite these methodological improvements, the consistency of impairment values across editions demonstrates that the 2024 updates enhance rather than disrupt the rating process. This balance of stability and innovation reinforces the AMA Guides’ role as the standard for impairment evaluation and supports a confident transition to the 2024 edition. Ultimately, the revision represents an evolution rather than a departure, preserving the integrity of the Guides while improving efficiency, interrater reliability, and consistency, strengthening stakeholder trust in its enduring role as the internationally recognized benchmark for objective impairment assessment.

## Figures and Tables

**Table 1:** The 2024 Five-Step Process for Lower Limb Impairment Rating.

Step 1.	Confirm a Clinically Relevant Diagnosis (DX)
Step 2.	Confirm Maximum Medical Improvement (MMI)
Step 3.	Identify the Relevant Diagnosis-Based Impairment (DBI) Table
Step 4.	Determine the Diagnostic Row, Class, Grade, and Impairment Value
Step 5.	Guidelines for Report Documentation

**Table 2:** Vignette Number and Associated Diagnosis.

Vignette	Diagnosis
16-01	Contusion Right Foot
16-02	Plantar Fasciitis
16-03	Ankle Instability
16-04	Bimalleolar Fracture
16-05	Ankle Arthritis
16-06	S/P Total Ankle Replacement with Poor Results
16-07	Knee Strain
16-08	Meniscal Tear
16-09	S/P Anterior Cruciate Reconstruction and Medial Meniscus Repair
16-10	Subluxing Patella
16-11	S/P Total Knee Replacement with Apportionment
16-11b	Knee Arthritis Apportionment Option
16-12	Knee Arthritis
16-13	Contusion Resolved Hip
16-14	Hip Dislocation and Relocation
16-15	Hip Subtrochanteric Fracture
16-16	Femoral Neuropathy
16-17	Complex Regional Pain Syndrome
16-18	Midfoot Amputation
16-19	Knee Motion Deficit
16-20	Amputation of Great Toe, Ankle, Knee
16-20a	Great Toe Amputation MTP joint
16-20b	Ankle Fracture Distal Fibula
16-20c	Knee ACL

**Table 3:** Comparison of Expert Evaluators’ Impairment Rating, 2008 vs. 2024.

	Lower Limb		Whole Person		2024 vs. 2008 Outcome Comparison	
Vignette	2024	2008	2024	2008	Lower Limb	Whole Person
16-01	0	0	0	0	Same	Same
16-02	1	1	1	1	Same	Same
16-03	5	5	2	2	Same	Same
16-04	15	20	6	8	Less by 5	Less by 2
16-05	30	26	12	10	More by 4	More by 2
16-06	59	59	24	24	Same	Same
16-07	0	0	0	0	Same	Same
16-08	1	1	1	1	Same	Same
16-09	12	12	5	5	Same	Same
16-10	16	16	6	6	Same	Same
16-11	31	31	12	12	Same	Same
16-11b	16	16	6	6	Same	Same
16-12	52	50	21	20	More by 2	More by 1
16-13	0	0	0	0	Same	Same
16-14	3	3	1	1	Same	Same
16-15	30	30	12	12	Same	Same
16-16	9	9	4	4	Same	Same
16-17	40	38	16	15	More by 2	More by 1
16-18	45	45	18	18	Same	Same
16-19	30	30	12	12	Same	Same
16-20	22	24	9	10	Less by 2	Less by 1
16-20a	12	13	5	5	Less by 1	Same
16-20b	0	0	0	0	Same	Same
16-20c	7	7	3	3	Same	Same
Combined Average Rating	18.17	18.17	7.33	7.29		
Average Difference	0.00		0.04			
Number of Vignettes Same					17	18
2024 Difference by More					3	3
2024 Difference by Less					3	2

**Table 4:** Mean Impairment Rating, 2008 vs. 2024.

Level (No. of Vignettes)	2024	2008	*p*-Value for Difference
Lower Limb (23)	18.17	18.17	0.98
Whole Person (23)	7.33	7.29	0.99
Average	12.75	12.73	0.92

**Table 5:** Comparison of the 2008 and 2024 AMA Guides Sixth Edition for Lower Limb Impairment.

Aspect	2008 Edition	2024 Edition
Framework	Impairment grids with multiple modifiers; less intuitive starting point	Diagnostic row framework anchored in specific clinical diagnoses
Clinical Integration	Less direct use of clinical history, exam, and studies	Direct integration of clinical history, exam, and relevant studies
Usability	Complex tables, more difficult to navigate	Simplified, transparent tables; easier to apply consistently
Consistency	Lower reproducibility; more evaluator variability	High inter-rater agreement; stronger reliability
Stakeholder Confidence	Impairment values less transparent, harder to justify	Clearer justification of values; greater acceptance by insurers and adjudicators
Patient-Centered Relevance	Limited direct linkage to patient function	Aligned with patient-centered management and meaningful outcomes
Future Orientation	Rigid structure, less adaptable to evolving practice	Adaptable framework encouraging ongoing research and updates

## Data Availability

The original contributions presented in this study are included in the article. Further inquiries can be directed to the corresponding author.

## Abbreviations

The following abbreviations are used in this manuscript:

AMA	American Medical Association
DBI	Diagnostic-based Impairment
